# The Reality of Neandertal Symbolic Behavior at the Grotte du Renne, Arcy-sur-Cure, France

**DOI:** 10.1371/journal.pone.0021545

**Published:** 2011-06-29

**Authors:** François Caron, Francesco d'Errico, Pierre Del Moral, Frédéric Santos, João Zilhão

**Affiliations:** 1 Institut National de Recherche en Informatique et en Automatique Bordeaux Sud-Ouest, Institut de Mathématiques de Bordeaux, Université de Bordeaux, Talence, France; 2 Unité Mixte de Recherche 5199 De la Préhistoire à l'Actuel: Culture, Environnement et Anthropologie, Université de Bordeaux, Talence, France; 3 Institute for Archaeology, History, Cultural and Religious Studies, University of Bergen, Bergen, Norway; 4 Seminari d'Estudis i Recerques Preistòriques, Universitat de Barcelona/Instituciò Catalana de Recerca i Estudis Avançats, Barcelona, Spain; University of Kansas, United States of America

## Abstract

**Background:**

The question of whether symbolically mediated behavior is exclusive to modern humans or shared with anatomically archaic populations such as the Neandertals is hotly debated. At the Grotte du Renne, Arcy-sur-Cure, France, the Châtelperronian levels contain Neandertal remains and large numbers of personal ornaments, decorated bone tools and colorants, but it has been suggested that this association reflects intrusion of the symbolic artifacts from the overlying Protoaurignacian and/or of the Neandertal remains from the underlying Mousterian.

**Methodology/Principal Findings:**

We tested these hypotheses against the horizontal and vertical distributions of the various categories of diagnostic finds and statistically assessed the probability that the Châtelperronian levels are of mixed composition. Our results reject that the associations result from large or small scale, localized or generalized post-depositional displacement, and they imply that incomplete sample decontamination is the parsimonious explanation for the stratigraphic anomalies seen in the radiocarbon dating of the sequence.

**Conclusions/Significance:**

The symbolic artifacts in the Châtelperronian of the Grotte du Renne are indeed Neandertal material culture.

## Introduction

For most of the 20th century, personal ornaments, systematic pigment use and elaborate bone technology were associated with the co-emergence of “Cro-Magnon” people and the Upper Paleolithic (beginning in Western Europe with the Châtelperronian) [1]. Over the last three decades, a number of findings challenged this view [2]–[9], namely: (a) in Africa, ornaments, pigments and abstract decoration occur among the immediate ancestors of modern humans; (b) anatomical modernity emerged earlier in Africa; (c) burial ritual, jewelry and body painting are known among Middle Paleolithic European Neandertals; (d) where the Châtelperronian is found with diagnostic fossils, these are of Neandertals, not modern humans; (e) the earliest evidence for anatomical modernity in Europe post-dates by many millennia the emergence of the Châtelperronian, corroborating the Neandertal authorship of the latter; (f) no sudden change or accretion is observed in the early stages of symbolic material culture but rather a discontinuous pattern of asynchronous emergence, disappearance and re-emergence of its features among both ‘modern’ and ‘archaic’ populations of the two continents. As a result, most paleoanthropologists now acknowledge that “symbolic thinking” and “modern behavior” are not species-specific features of anatomically modern humans and that Neandertals were the makers of a symbolic material culture [4], [7]–[13].

**Figure 1:**
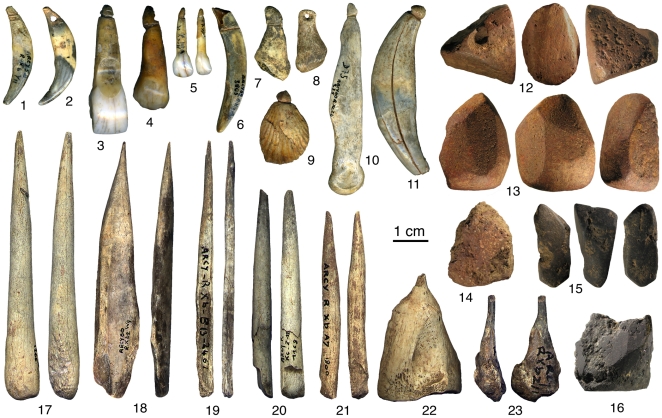
Grotte du Renne, Châtelperronian symbolic artifacts. Personal ornaments made of perforated and grooved teeth (1–6, 11), bones (7–8, 10) and a fossil (9); red (12–14) and black (15–16) colorants bearing facets produced by grinding; bone awls (17–23). 1–9 courtesy of M. Vanhaeren, 12–16 courtesy of H. Salomon, 17–23 modified after [24].

The Châtelperronian levels (VIII, IX and X) of a French cave site, the Grotte du Renne (Arcy-sur-Cure, Burgundy), document the association between Neandertal remains and large numbers of personal ornaments, bone tools (some of which are decorated), and colorants (Figure 1). Although excavated (by A. Leroi-Gourhan) between 1949 and 1963 [14]–[15], the techniques used were modern (stratigraphic digging and area exposure of occupation surfaces, spatial plotting of key finds and features, systematic sieving of the deposits), and the geological reality of the described succession—confirmed by limited excavation of a stratigraphic baulk carried out in 1998 [16]—is uncontroversial. Even so, the following concerns have been voiced with respect to the homogeneity of the artifact assemblages [17]–[20]: (a) no investigation of potential refitting of stone tools across levels has been carried out, so the extent to which the Grotte du Renne sequence was affected by post-depositional disturbance cannot be assessed; (b) the existence of overlying Protoaurignacian, Aurignacian/Gravettian and Gravettian levels (VII, VI and V, respectively) and the fact that some ornament types present in the Châtelperronian sequence (e.g., pierced fox teeth) are common in later Early Upper Paleolithic (EUP) technocomplexes raise the possibility that the symbolic artifacts found in the Châtelperronian are intrusive from above; (c) conversely, as the habitation structures built by the first Châtelperronian occupants of the site conceivably disturbed the underlying Middle Paleolithic levels (XI–XIV), the Neandertal remains recovered in levels VIII–X could be intrusive from below.

On the basis of these concerns, three alternatives to the stratigraphic integrity of the Châtelperronian levels of the Grotte du Renne are conceivable: (a) the personal ornaments, bone tools and colorants are Protoaurignacian or later and the Neandertal remains are Mousterian, so the site's Châtelperronian lacked symbolic artifacts and is of unknown authorship (hereafter Hypothesis 1); (b) the personal ornaments are Protoaurignacian or later, so the site's Châtelperronian, although made by Neandertals, lacked symbolic artifacts, as the colorants and bone tools may be regarded as purely functional (hereafter Hypothesis 2); and (c) the personal ornaments, bone tools and colorants are Châtelperronian, but the Neandertal remains are Mousterian, so the Châtelperronian may well have been made by modern humans instead (hereafter Hypothesis 3).

In the following, the distributions of ornaments, bone tools, colorants, pigment processing tools and human teeth are compared with those of diagnostic stone tools: Levallois flakes (Mousterian); Châtelperron points and convergent sidescrapers, the latter as a proxy for the *racloir châtelperronien*, a tool type that Leroi-Gourhan [21] defined as characteristic of the site's Châtelperronian but is not discriminated as such in available inventories; Dufour bladelets and their unretouched blanks (Protoaurignacian). Using this tested taphonomic approach [22], we assess whether the final condition represented by the observed distributions can be derived by post-depositional disturbance from any of the initial conditions implied by Hypotheses 1–3, which, statistically, are our null hypotheses. Our results reject them. Therefore, the alternative view—the association between symbolic artifacts, Châtelperronian stone tools and diagnostic Neandertal fossils characteristic of levels VIII–X is genuine and reflects the behavior of the Grotte du Renne's Neandertal occupants—stands unrefuted.

## Results

Of the different post-depositional processes that can affect an archeological site [23], generalized, large scale disturbance can be rejected from further consideration in this case because the diagnostic artifacts, upon which the different levels were assigned to different technocomplexes, would have become scrambled and the sequence would have been recognized from the outset as stratigraphically mixed. As shown by the distributions for levels VII–XIV (Table 1), nothing can be farthest from the truth, as 100% of all Levallois flakes were recovered in Mousterian levels XI–XIV, 99% of all Châtelperronian points were recovered in Châtelperronian levels VIII–X, and 100% of the bladelets (both Dufour bladelets and unretouched blanks) were recovered in Protoaurignacian level VII.

**Table 1:** Grotte du Renne. Stratigraphic distribution of key finds*.

Levels	Ornaments	Pigments	Workedbone	Neandertalteeth	Dufourbladelets	Châtelperronpoints	Levalloisflakes	Unretouchedbladelets	Convergentsidescraprers
VII	8	39	70	0	287	1	0	2800	0
VIII	8	146	27	1	0	29	0	0	2
IX	2	286	17	3	0	67	0	0	28
X	29	1183	139	25	0	284	0	0	105
XI	0	5	1	1	0	2	9	0	4
XII	0	4	1	1	0	0	14	0	2
XIII	0	0	0	0	0	0	3	0	1
XIV	0	0	0	3	0	0	0	0	2

*In order to simplify, and also because inventories of the different find categories are unavailable for them, levels VI and above are not considered here.

Conceivably, two other mechanisms could have created the notional contradiction between a stratigraphic distribution of lithic diagnostics that matches expectations in >99% of the instances and the putative displacement of entire categories of items found in association with such diagnostics [23]. One such mechanism is localized, large scale displacement reaching non-adjacent levels of the stratigraphy (e.g., as a result of mammal burrowing or subsurface anthropogenic intervention); the other is small scale, gradual and cumulative displacement of individual objects across the entire sequence through geological or pedogenetic processes (e.g., as a result of cryoturbation or root/worm activity).

### Localized, large scale displacement

The stratigraphic consistency of vertical distributions does not counter, per se, that unrecognized disturbance of a particular area of a given level may explain the presence in that stratigraphic unit of find categories otherwise absent from intact areas. For instance, in the case of Châtelperronian level X of the Grotte du Renne, the ornaments and other symbolic items could correspond to a subsurface cache created by level VII Protoaurignacian people that burrowing animals subsequently moved further down, while the Neandertal remains could come from a discrete accumulation (related to e.g. secondary burial) that Châtelperronian construction activity or animal burrowing subsequently moved up from Mousterian levels XI–XII.

**Figure 2:**
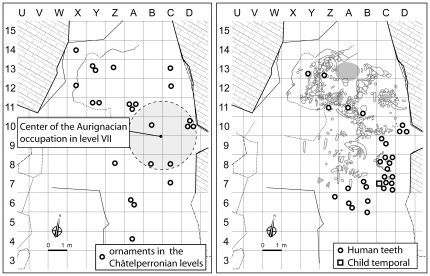
Grotte du Renne distributions. Left: personal ornaments from Châtelperronian levels VIII–X plotted against the most densely occupied area of Protoaurignacian level VII. Right: Neandertal remains from Châtelperronian levels VIII–X plotted against the habitation features in level X. The areas in grey are hearths.

**Figure 3:**
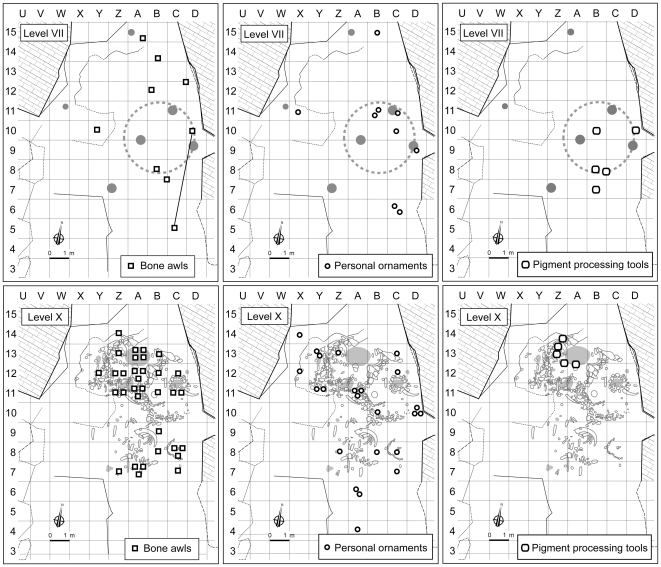
Grotte du Renne, distribution of bone awls, ornaments and pigment processing tools. Top: in Protoaurignacian level VII. Bottom: in Châtelperronian level X. The areas in grey are hearths.

**Figure 4:**
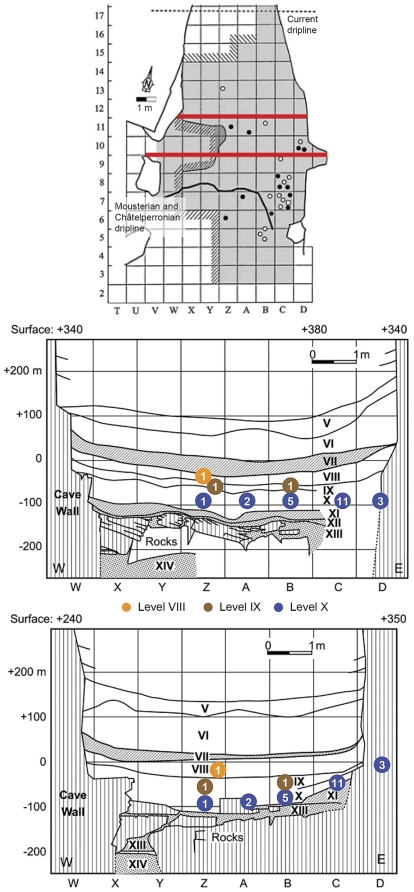
Distribution of the Neandertal remains in Châtelperronian levels VIII–X of the Grotte du Renne; after [**5]**, [19****], modified. In the plan (above), the black circles are diagnostic teeth, the white circles are teeth whose features are consistent with (but do not prove) assignment to the Neandertals, the white square is the immature temporal bone, and the red lines indicate the position of the stratigraphic profiles shown middle and bottom.

The expected outcome of the displacement of such find clusters is a pattern where the original concentration, even if diluted by the disturbance process, would be preserved to some extent, defining a scatter with a clear center close to its original location and increasingly sparse toward the periphery. In contrast to such an expectation, the personal ornaments in level X feature a homogeneous, low density distribution across the entire excavated surface, with most finds coming from outside the area of densest Protoaurignacian occupation, while the bone awls [24] form two broad clusters that coincide with the location of the Châtelperronian habitation features (Figures 2,3). The Neandertal remains are also scattered and, although found outside the habitation features, were recovered well inward of the dripline, countering the notion that they reflect dumping at the cave entrance of Mousterian sediments removed by Châtelperronian construction activity (Figure 4). The apparent concentration against the East wall reflects the presence of three groups of teeth from three different individuals, interpreted as the in situ disintegration of single mandibular or maxillary pieces [5].

Another hypothesis is that the Neandertal teeth derived by progradation from stratigraphically lower but topographically higher Mousterian deposits [19]. Conceivably, this could have happened southward of row 8 and westward of row C, because of the marked slope of the strata outside the Mousterian dripline. Most teeth (80%), however, were found inward of this dripline, where the stratification of the Châtelperronian deposits is horizontal. Most were also found against the eastern wall of the cave—14 out of the 22 securely provenanced to level X (64%) came from rows C–D of the grid (Figure 4). Given this spatial distribution, the presence of Neandertal teeth in level X cannot result from progradation processes associated with the westward dip of immediately underlying Mousterian level XI. Such processes are gravity-driven, so they could have diplaced material into the adjacent Y-B rows lower down but not into the exact same C–D rows higher up. Rather than the 25 Neandertal teeth in Châtelperronian level X reflecting downslope erosion of the Mousterian deposits towards the center of the cave, it is the two teeth in Mousterian levels XI–XII that likely correspond to downward displaced items, as is the case with the few bone awls and Châtelperron points also found in levels XI–XII [7], [24].

**Figure 5:**
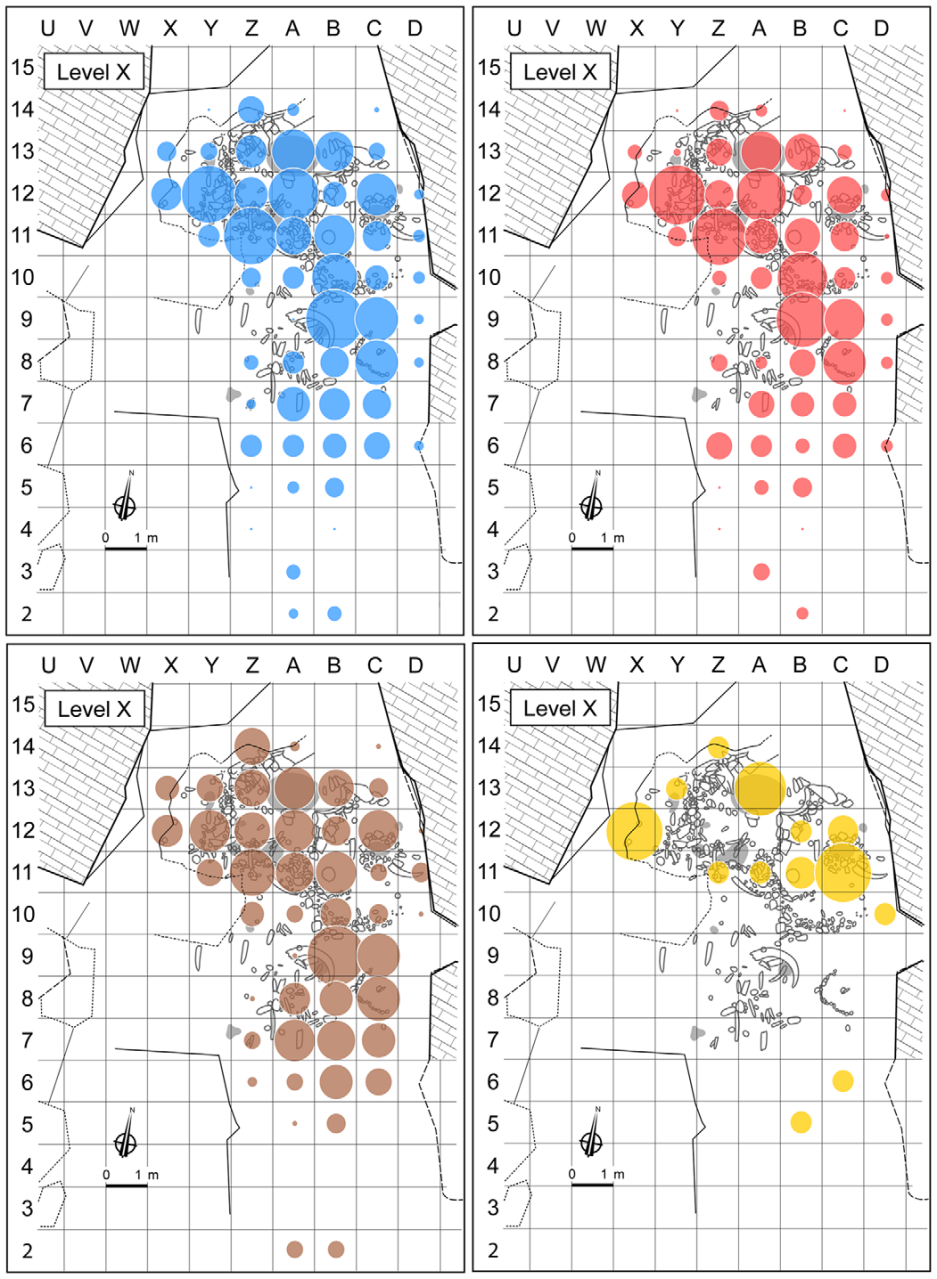
Spatial distribution of colorants by weight in Châtelperronian level X (all sublevels included); data from [**27**] **.** Top left, all colors (14,580 g); top right, red (10,520 g); bottom left, black (3,961 g); bottom right, yellow (99.6 g). Bubble sizes reflect the relative frequency per grid unit and were calculated by assigning the following values to the four weight classes given in the data source: [0.1–10 g]  = 10 g; [10–50 g]  = 50 g; [50–100 g];  = 100 g; [>100 g]  = 150 g. Major concentrations are apparent despite the smoothing of the distributions caused by the quantification procedure and they coincide with the habitation features. The grey areas are hearths.

The distribution of ornaments, bone tools, pigments and pigment processing tools [25]–[29] is congruent with the placement of the habitation features recognized (Figures 3, 5). In Protoaurignacian level VII, there is a concentration on the eastern side of the cave, almost exclusively in bands B–D, where the few pigments and pigment processing tools were associated with hearths (a large spot of red pigment was also noted toward the entrance). In Châtelperronian level X, the spatial distribution of the abundant pigments changes slightly between sublevels but, in agreement with the location of the tools for pigment processing, maximum concentrations systematically occur inside the features identified in the central and northwestern parts of the cave, where most ornaments and bone tools were also found. These patterns strengthen the case for the stratigraphic integrity of levels VII and X and further counter the notion that the symbolic items found therein were differentially affected by post-depositional disturbance.

### Generalized, small scale displacement

Limited post-depositional movement across the boundaries of adjacent levels is a ubiquitous feature of cave and rockshelter stratigraphies. The Grotte du Renne is unlikely to have been immune to such processes, and excavation error can result in the misassignment of finds from the interface between different units. Indeed, as with the two awls in the Mousterian that probably come from Châtelperronian level X, four small ornamental ivory fragments from uppermost Châtelperronian level VIII probably originated in the immediately overlying Protoaurignacian [7], [30].

Do such ordinary post-depositional mechanisms suffice to support any of the Hypotheses 1–3 above? Intuitively, no, because (a) explaining the “advanced” finds made in the Châtelperronian levels as entirely intrusive from the later EUP in levels VII or above faces the problem that most such finds came from level X, not level VIII, and (b) although most Neandertal remains from the Châtelperronian were in level X, the notion that adjacent Mousterian levels XI–XII seeded the fossils recovered in the immediately overlying unit faces the problem that only 6% would have remained put [7]–[8], [30]–[31].

In order to more rigorously test whether the operation of this mechanism could be held responsible for the observed distributions, we modeled it as a random process whereby the final condition (Table 1) derives from the initial condition (Table S1) as a result of small scale displacements affecting the entire thickness of the deposits concerned here (see Materials and Methods). In each case, the results of the goodness of fit test reject, for a probability threshold of 0.01, the possibility that the observed distribution can be the outcome of the disturbance, via the modeled mechanism, of a sequence presenting any of the original distributions implied by Hypotheses 1–3. Under Hypotheses 1 and 2, the distribution of the items putatively introduced from level VII or above would have to feature a gradual decrease with depth instead of the observed marked concentration in level X (which is all the more remarkable in the colorants' case as, by weight, the observed totals are 0.45 kg in level VII and 14.58 kg in level X; Table 2). Likewise, under Hypothesis 3, the upwardly displaced Neandertal remains would have to be in much smaller numbers than those remaining in situ. Finally, under any of the hypotheses, the displacements would significantly affect the diagnostic stone tool types: under Hypotheses 1 and 2 it is extremely unlikely than no bladelets accompanied the downward displacement of the ornaments and under Hypothesis 3 it is extremely unlikely that no Levallois flakes accompanied the upward displacement of the Neandertal remains.

**Table 2:** Stratigraphic distribution of colorants (by weight, in grams) at Grotte du Renne; data from [27].

Levels	Cultural Attribution	Red	Black	Yellow	Total	%
V	Gravettian	1179.4	14.5	13.9	1207.8	6.0
VI	Aurignacian/Gravettian	196.5	1.5	147.2	345.2	1.7
VII	Protoaurignacian	404.4	29.5	17.3	451.2	2.2
VIII	Châtelperronian	966.2	231.7	28.2	1226.1	6.1
IX	Châtelperronian	1749.3	476.4	17.2	2242.9	11.1
X	Châtelperronian	10,520.2	3961	99.6	14,580.8	72.4
XI	Mousterian	48.5	17	12	77.5	0.4
TOTAL		15,064.5	4731.6	335.4	20,131.5	100.0

Assuming generalized, small scale disturbance, the worst case scenario for the stratigraphic integrity of the Grotte du Renne is one where, against the geological evidence, the three Châtelperronian levels (VIII–X) are conflated into a single occupation, and post-depositional displacement is modeled as occurring between two adjacent blocks only—for the ornaments, involving overlying Protoaurignacian level VII, and, for the Neandertal teeth, involving underlying Mousterian levels XI–XII (see Materials and Methods). Even so, the observed distribution of the lithic diagnostics implies that, for the predicted initial distributions (Table S1) and a probability threshold of 0.01, no more than 1 ornament (Hypothesis 2) or 7 Neandertal teeth (Hypothesis 3) could have found their way into that single Châtelperronian block (Figure S3), whereas the observed totals are 39 and 29, respectively.

## Discussion

Since the observed vertical and horizontal distribution patterns cannot be derived by post-depositional disturbance from the initial set of conditions implied by Hypothesis 1–3, we conclude that the association between Châtelperron points, personal ornaments, bone tools, colorants and Neandertal remains found at the Grotte du Renne is genuine. In contrast, using Bayesian statistics applied to the stratigraphic distribution of bone tools and human-modified faunal remains directly dated by radiocarbon, a recent assessment of the site's integrity argued for significant problems because more than one third of the dates turned out to be outliers [20].

Our results show that the reason for this anomaly must be sought elsewhere, as the distribution of the lithic diagnostics sets strict limits to the potential disturbance of the other find categories, including those addressed by the radiocarbon study and not considered here (such as the faunal remains). For instance, 8 (out of 21) Châtelperronian samples came out younger than or overlapping with the ∼36.5 ka ^14^C BP limit set by chronostratigraphic patterns for the beginning of the Protoaurignacian in Europe [32]. But, since 0 out of 287 Dufour bladelets and 0 out of 2800 unretouched bladelets intruded, (a) the probability that those 8 samples correspond to intrusive items is <1e-18 and (b) the probability that more than one dated Châtelperronian sample is displaced from level VII is <0.01 (Figure S3). Given these probabilities, it is clear that the accuracy of the results obtained should be treated as an open issue, which invalidates the outlier test—in this context, a simple conclusion of the premise that the radiocarbon results are accurate.

Under this light, the Grotte du Renne's radiocarbon dating history is very instructive. Given that, in agreement with predictions made on the basis of archeological criteria [32], the new ages for Protoaurignacian level VII fell in the ∼34.5–36.5 ka ^14^C BP range, the underlying Châtelperronian levels VIII–X should be chronometrically older. However, only 2 out of the 17 results (12%) previously published for the Châtelperronian of the Grotte du Renne satisfied such a condition [20], which is the case for at least 13 of the new samples (62%). This progress can only have come from ameliorated measurement and pretreatment techniques (e.g., ultrafiltration) [33], not from the site's stratigraphic integrity having improved over the years. In this context, the parsimonious explanation for why a minority of the new results came out younger than expected should be based on (a) the poor preservation of collagen at the site, as reported [20], and (b) the incomplete decontamination of a number of samples.

The impact that even trace amounts of contaminants have on samples whose ages are close to the limit of applicability of radiocarbon is huge. For instance, if a sample is 40,000 years old, as little as 0.6% of a contaminant dated to the time of the excavation of the finds, 50 years ago, would suffice to produce a measured age of 35,000. The Grotte du Renne's faunal remains and bone tools were extensively preserved with glues and consolidants, and indeed this was the single site among those dated by the Oxford-led project to assess the chronology of the Middle-to-Upper Paleolithic transition in Europe [34] where such kinds of samples, otherwise rejected as unsuitable, were used—in fact, at the Grotte du Renne, they represent as much as 84% of the determinations.

**Figure 6:**
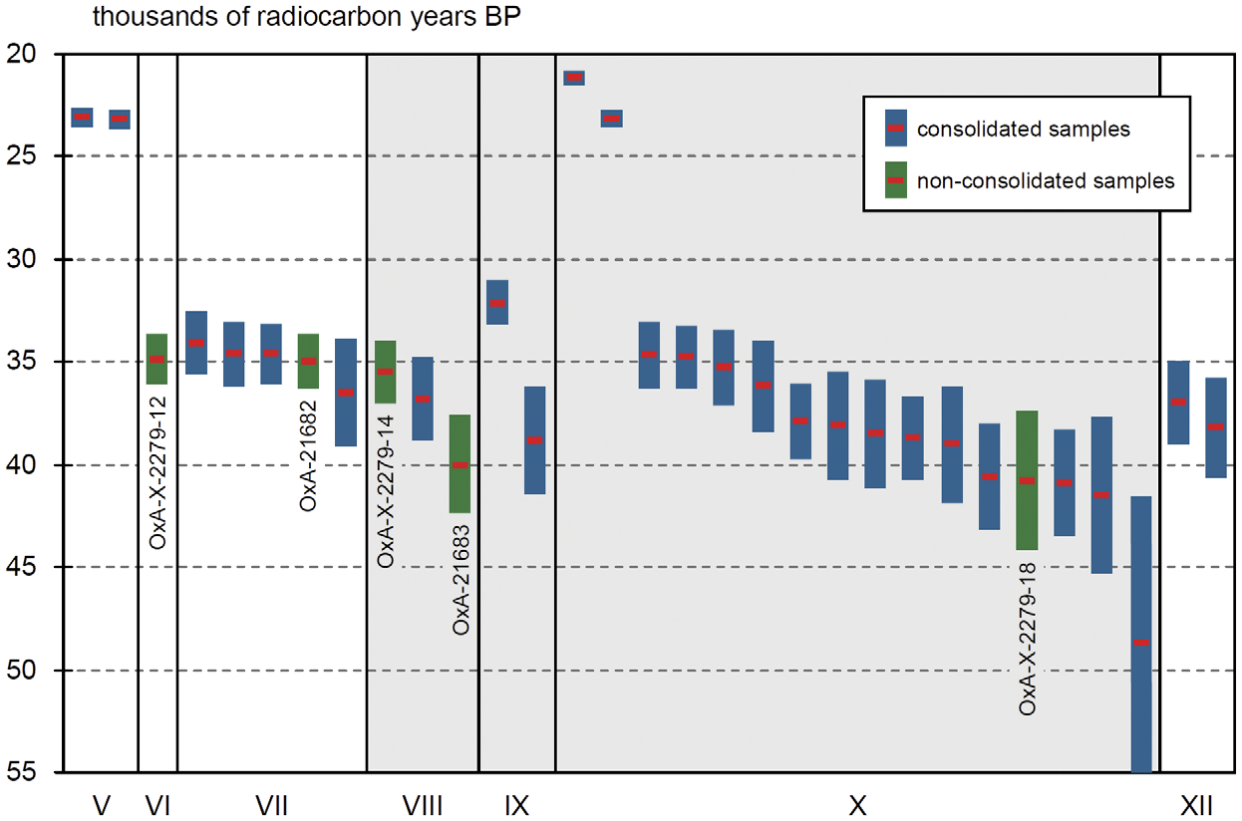
Plot of age (mean value and 2σ confidence interval) against stratigraphic provenience of the new radiocarbon dates for the Grotte du Renne; data from [**20**]. Levels VIII–X are Châtelperronian.

To circumvent the problems of potential contamination the radiocarbon study used a solvent sequence phase of pretreatment before collagen extraction whenever visual inspection indicated curatorial preservation. In one case, Poly-Vinyl Acetate (PVA) was identified. This ^14^C-free material cannot be the cause for results younger than expected but the study also found that it was impossible to rule out trace contamination from this or other sources. That some remained indeed is indicated by (a) the C:N value of 3.7 associated with one of the Châtelperronian results (OxA-X-2226-7), which the study accepted despite acknowledging that it indicated the presence of exogenous carbon, (b) the C:N value of 3.6, also above the OxA threshold for acceptance, obtained for a sample from level X dated to ∼23.1 ka ^14^C BP (OxA-X-2222-21), (c) the vertical distribution of the few dates for unconsolidated samples, which are fully consistent with stratigraphy and, at the 95.7% confidence level, with the ∼36.5 ka ^14^C BP boundary for the Protoaurignacian (Figure 6), (d) the fact that outliers only occur in samples from level IX or below (all dates for uppermost Châtelperronian level VIII and those above correlate perfectly with stratigraphic depth), and (e) the results for Mousterian level XII, younger than those obtained for well dated occurrences of the Châtelperronian elsewhere in France, namely at Roc-de-Combe and the eponymous site of the Grotte des Fées [8], [22].

These level XII results play a critical role in the outlier analysis underpinning reference [20] 's interpretation that the Grotte du Renne sequence is significantly disturbed. In the framework of the Bayesian approach followed, the results for level XII constrain the age of the overlying units, for instance making the date of 48,700±3600 ^14^C BP (OxA-X-2279-44) for level X appear too old for its stratigraphic position and, hence, as an intrusion form the underlying Mousterian. In fact, when that ∼48.7 ka ^14^C BP result is statistically compared one-to-one with those for the same level accepted by the outlier analysis (for instance, using the tools in the Calib 6.0 software, http://calib.qub.ac.uk/calib/), all come out as identical.

More importantly, accepting the outcome of the outlier analysis should have invalidated the use of Bayesian modeling to reconstruct the chronology of the site—if significant movement across levels occurred, the stratigraphic position of a given sample cannot be used to constrain the probability distribution associated with its determined age. This is because, in that case, the real chronological order of the samples, an a priori requirement of the modeling, must be considered unknown—once significant post-depositional displacement is posited, stratigraphic provenance cannot be taken as an indicator of relative age. For instance, in Figure 2 of reference [20], the two level XII samples are used to define the lower chronological boundary of the Châtelperronian but this assumes that the samples are in situ. However, if, as the authors claim, as much as 30% of their 31 determinations are outliers and this implies post-depositional disturbance, then we cannot reject the possibility that those samples represent instead intrusions from overlying Châtelperronian X, as otherwise suggested by the presence of a Châtelperronian bone awl in that level [24].

To be consistent with the results of the outlier analysis, the start of the sequence in reference [20] 's age model should be given by the ∼48.7 ka ^14^C BP result for level X because, if the latter is statistically distinct from the others from the same level and deemed to be stratigraphically displaced, then it can only have been displaced from the Mousterian. This carries the implication that the beginning of the Mousterian phase should be represented by that ∼48.7 ka ^14^C BP result, not, as assumed by the model, by the two results in the ∼37–38 ka ^14^C BP range obtained for level XII, which, in turn, significantly decreases the number of outliers. The distortion imposed on the data by reference [20] 's unwarranted use of Bayesian modeling also impacts the estimated duration of the sequence. This is readily apparent when comparing its figures 2 (modeled calibrated dates) and S2 (unmodeled calibrated dates): in the former, the succession of levels XII to VI spans some 5000 years, while the latter suggests that it may in fact correspond instead to as much as 10,000.

Given the above, the interpretation of the anomalously young results for the Grotte du Renne should be identical to that for similary consolidated bone samples whose collagen was similarly poorly preserved and whose sample chemistry similarly failed to meet all the standards of the (same) dating laboratory—namely, the two Neandertal bones from Vindija dated at Oxford, which treated them as minimum ages only [35]. But even if the accuracy of all the new radiocarbon results for the Grotte du Renne is accepted, it can be calculated (given the 38% putatively displaced Châtelperronian samples this implies, and assuming that all finds are equally susceptible to displacement), for a probability threshold of 0.01, that at least 8 of the 39 ornaments and 5 of the Neandertal teeth in Châtelperronian levels VIII–X cannot be intrusive (Text S1). Obviously, this calculation ignores the modeling boundaries imposed by the distribution of the diagnostic stone tools, as if their cultural and chronological attributes were meaningless and the Châtelperronian and Protoaurignacian labels void to begin with. The point, however, is that even this unrealistic scenario fails to reject that at least a part of the Grotte du Renne's Neandertal teeth and symbolic artifacts are truly in situ.

Finally, we note that recent re-analysis of the Quinçay rockshelter, the other major Châtelperronian site featuring ornaments (six pierced teeth of deer, fox and wolf), showed that there is little ground to question its stratigraphic integrity [36]–[37]. A small component of retouched bladelets is found in all levels but in association with the cores whence the corresponding blanks were extracted and those cores are of typical Châtelperronian technique. This material cannot represent intrusion from overlying Aurignacian levels, which are non-existent at Quinçay, where the Châtelperronian sequence was sealed by collapsed limestone slabs several meters long and ∼50 cm thick.

### Conclusion

Our results reject the notion that the association of symbolic artifacts with Neandertals at the Grotte du Renne results from large scale localized or small scale generalized displacement of artifacts and human remains. They imply that the parsimonious explanation for the anomalies observed in the radiocarbon dating of the sequence is incomplete decontamination of the bone samples used. Moreover, the Grotte du Renne is not alone. Similar, and even earlier evidence of Neandertal symbolically mediated behaviors has now been produced for the Middle Paleolithic of Iberia and Italy [9], [38], and there is sufficient ground to postulate that coeval material from the French Châtelperronian, the Italian Uluzzian [39], and a number of sites in Central and Western Europe with less clear-cut stratigraphic patterns [30], [31] is also Neandertal-associated. While recent Grotte du Renne research developments have been claimed to herald the bursting of the Neandertal ornament “bubble” [40], our results and their wider context show that such news were “greatly exaggerated.” The Châtelperronian levels of the Grotte du Renne do stand for Neandertal symbolism.

## Materials and Methods

To assess post-depositional displacement of human remains we used loose teeth (given their small size and mobility and the fact that, except for a temporal fragment, no other human skeletal parts were recovered at the Grotte du Renne), and only considered those unambiguously provenanced to a specific grid unit. In ornament counts, fragments of possibly the same object were considered separately. These criteria explain the minor differences with previously published inventories [8], [30], [31]. We used a continuous time model positing that the different items can move from one level to the next at a given rate, which we assume to be the same for both the direction of the movement (upward or downward) and the category concerned because no significant difference in physical properties (size, weight or density) exists that justifies discriminating in this regard between, say, a Dufour bladelet, a Neandertal tooth or a pierced fox canine. From top to bottom, and using available radiometric information [20], we set the upper time limits for the formation of the different stratigraphic units at 40.5 (VII), 41.5 (VIII), 43.0 (IX), 44.5 (X), 46.0 (XI), 47.5 (XII), 49.0 (XIII) and 50.5 (XIV) thousand calendar years ago (but found the results of the exercise to be unchanged even if the age of the lower, undated units was moved back in time by several millennia). We then estimated, for each hypothesis, the rate parameter that best fitted, according to the Pearson chi square statistic [41], [42], the transformation between the initial and final conditions. This best fitted rate parameter provides the best trade-off regarding objects that have not moved (e.g. Dufour or unretouched bladelets) and objects that have moved (e.g. Neandertal teeth or ornaments) under the different hypotheses. We then conducted a goodness of fit test to assess the likelihood that the final distribution was generated from the hypothesized initial distributions (Table S1) via the model. Using the best fitted rate parameter for each hypothesis, we obtained expected final distributions for each find category and probabilities that any given item moved from any given level to another (Tables S2, S3). These distributions were then compared with the observed final distributions (Table 1) to assess the goodness of fit of the model associated with each hypothesis. This goodness of fit is measured by the Pearson chi square statistic, which provides a positive measure of discrepancy between the expected and true final distributions of objects. Having concluded that small scale, generalized displacement failed to account for significant movement in some find categories and none in others, we then calculated, using the Bayes theorem [43], the probability that items from the key find categories (Neandertal teeth and personal ornaments) had moved into the Châtelperronian from the overlying Protoaurignacian or the underlying Mousterian under the constraints posed by the distribution of diagnostic stone tools. A technical description of our statistical approach is provided in Text S1.

## Supporting Information

Text S1 **Hypothesis testing, continuous time model and probability models.**
(PDF)

Figure S1 **Realization from the continuous time model, for an object starting in level XI.**
(TIFF)

Figure S2 a: *S* in function of λ for Hypothesis 1; b: *S* in function of λ for Hypothesis 2, c: *S* in function of λ for Hypothesis 3.
(TIF)

Figure S3 a: probability that k ornaments over a set of 47 (the total number of ornaments from levels VII and VIII–X) are intrusive, given the number of intruded Dufour bladelets and unretouched bladelets; b: probability that k Neandertal teeth over a set of 31 (the total number of Neandertal teeth from levels XI–XII and VIII–X) have moved from levels XI–XII to levels VIII–X, given the number of Levallois flakes that have moved; c: probability that k samples over a set of 26 (the total number of dated samples from levels VII and VIII–X) are intrusive, given the number of intruded Dufour bladelets and unretouched bladelets.
(TIF)

Table S1 **Predicted initial distribution of finds for the different tested hypotheses.**
(DOC)

Table S2 **Expected final values** ***E*** **(rounded) associated to the best fitted λ for the different tested hypotheses.**
(DOC)

Table S3 **Values π associated to the best fitted λ for the different hypotheses.**
(DOC)
