# Supplementary material for: The Reality of Neandertal Symbolic Behavior at the Grotte du Renne, Arcy-sur-Cure, France
Source: PLoS One. 2011 Jun 29;6(6):e21545. doi: 10.1371/journal.pone.0021545 (PMC3126825; doi:10.1371/journal.pone.0021545)
Supplement: Text S1 — Hypothesis testing, continuous time model and probability models. (PDF) [file pone.0021545.s001.pdf]

The reality of Neandertal symbolic behavior at the Grotte du Renne, Arcy-sur-Cure

François CARON

Francesco D'ERRICO

Pierre DEL MORAL

Frédéric SANTOS

João ZILHÃO

Supporting Text S1

Continuous time model

The statistical model

Let $k=1,\ldots,K$ be the index for the different types of elements (ornaments, red pigments, etc.) (3). Let X_i^k be the initial number of elements of type k at level $i=1,\ldots,n$. Let Y_i^k be the actual number of elements of type k at level $i=1,\ldots,n$. In our case, $K=9$ (ornaments, pigments, worked bone, Neandertal teeth, Dufour bladelets, Châtelperron points, Levallois flakes, unretouched bladelets and convergent sidescraprers) and $n=8$ (VII, Protoaurignacian; VIII, IX and X, Châtelperronian; XI, XII, XIII and XIV, Mousterian). As an example, we have, for the ornaments $(Y_1^1,\ldots,Y_8^1)=(8,8,2,29,0,0,0,0)$ and, under Hypotheses 1 and 2, $(X_1^1,\ldots,X_8^1)=(47,0,0,0,0,0,0,0)$.

Let T_i be the time difference between level i and now. Consider an element starting at level i and ending in level j. We assume that at some random times t_1, t_2, etc. between 0 and T_i the element can move to an upper/lower level with equiprobability. We assume that the random times are distributed according to a Poisson process of unknown rate $\lambda>0$. Figure S1 shows a realization from this process, starting from level XI and ending in level XII.

For any element, the number of events (up or down) over a period T_i thus follows a discrete Poisson distribution of parameter λT_i. Let N^+ be the number of events where the element went to an upper level and N^- the number of events where the element went to a lower level, then we have

$$N^+ \sim Poisson(\frac{\lambda}{2}T_i) \quad (1)$$

$$N^- \sim Poisson(\frac{\lambda}{2}T_i) \quad (2)$$

where '~' means '*statistically distributed from*' and Poisson (λ) is the usual discrete Poisson distribution of parameter λ. For any given item, the difference between the ending level and the starting level is $N=j-i$ with $N=N^+-N^-$ and where N^+ is the number of times an item moved up and $N^-$is the number of times an item moved down. N is distributed from a Skellam distribution whose probability mass function is given, for $k\in\mathbb{Z}$, by

$$P(N=k)=\exp(-\lambda T_i)I_{|k|}(\lambda T_i) \quad (3)$$

where $I_k(z)$ is the modified Bessel function of the first kind. As we need to have $j=(i+N)\in\{1,\ldots,n\}$, we need to consider the truncated Skellam probability distribution. It follows that the probability π_{ij} to go from a level $i\in\{1,\ldots,n\}$ to a level $j\in\{1,\ldots,n\}$ over the time T_i for a single element is given by

$$\pi_{ij} = \frac{\exp(-\lambda T_i) I_{|j-i|}(\lambda T_i)}{\sum_{\ell=1}^{n} \exp(-\lambda T_i) I_{|\ell-i|}(\lambda T_i)} \tag{4}$$

For $i = 1, \ldots, n$, let $\pi_i = (\pi_{i1}, \ldots, \pi_{in})$.

Let Z_{ij}^k denote the number of items of type k that were in level i at the initial time and end up in level j at the final time (after T_i). As every item moves independently, we have, for $i = 1, \ldots, n$ and $k = 1, \ldots, K$

$$(Z_{i1}^k, \ldots, Z_{in}^k) \sim \mathsf{M}(\pi_i, X_i^k) \tag{5}$$

where $Z_i^k = (Z_{i1}^k, \ldots, Z_{in}^k)$ and $\mathsf{M}(\pi, X)$ denote the standard multinomial distribution of parameters π and X. The items observed at the final time are the sums of items coming from different levels, hence, for $j = 1, \ldots, n$ and $k = 1, \ldots, K$

$$Y_j^k = \sum_{i=1}^{n} Z_{ij}^k \tag{6}$$

Here is the pseudo-code to sample from the model:

• For $i = 1, \ldots, n$, compute π_{ij} as in Equation (4)

• For $k = 1, \ldots, K$

- for $i = 1, \ldots, n$, sample $Z_i^k \sim \mathsf{M}(\pi_i, X_i^k)$

- for $j = 1, \ldots, n$, let $Y_j^k = \sum_{i=1}^{n} Z_{ij}^k$

For this, we write simply, for $k = 1, \ldots, K$

$$(Y_1^k, \ldots, Y_n^k) \sim F_\lambda(X^k)$$

where F_λ is the distribution defined above, to emphasize the dependency with respect to λ.

Hypothesis testing

We want to perform a goodness of fit test to see whether the above model gives a good or a bad fit to the observed data. We assume that

$$Y^k \sim F_{true}(X^k)$$

where F_{true} is the true distribution of the data, and we want to test the hypotheses

$$H_0 : F_{true} = F_\lambda \text{ vs } H_1 : F_{true} \neq F_\lambda$$

To do so, we can consider the classical Pearson chi square statistic, defined by

$$S = \sum_{k=1}^{K} \sum_{j=1}^{n} \frac{(Y_j^k - E_j^k)^2}{E_j^k} \tag{7}$$

where

$$E_j^k = \mathrm{E}[Y_j^k]$$

$$= \sum_{i=1}^{n} \mathrm{E}[Z_{ij}^k]$$

$$= \sum_{i=1}^{n} X_i^k \pi_{ij}$$

Under H_0, the test statistic is approximately chi square distributed with $K(n-1)$ degrees of freedom. For a given size α, we can compute a threshold Th_α such that we reject H_0 if $S > Th_\alpha$.

Parameter estimation

We need to estimate the rate parameter $\lambda > 0$. To do so, we can choose the value that minimize the test statistic S. Optimization can be done over a one-dimensional grid.

Summary

The whole strategy is described below

- Parameter estimation
- For each value of λ

  (a) for $i = 1,\ldots,n$, $j = 1,\ldots,n$ compute π_{ij}

  (b) for $j = 1,\ldots,n$ and $k = 1,\ldots,K$, compute $E_j^k = \sum_{i=1}^{n} X_i^k \pi_{ij}$

  (c) compute $S = \sum_{k=1}^{K} \sum_{j=1}^{n} \frac{(Y_j^k - E_j^k)^2}{E_j^k}$

- Hypothesis testing
- Let S_{min} be the minimum value obtained. If $S_{min} > Th_\alpha$, then reject H_0.

Results

The final (observed) conditions are given in Table 1. The initial conditions for the different hypotheses are given in Table S1, and the values of $S(\lambda)$, computed over a grid of values for λ, are reported in Figure S2.

For Hypothesis 1, the minimal value $S = 15331.55$ is obtained for $\lambda = 0.000124$. It means that an element will move to an upper/lower level every $\frac{1}{\lambda} = 8065$ years on average (the distribution between time events indeed follows an exponential distribution with this parameter). The quantities π and E associated to this best fitted value are given in Tables S2-S3, respectively. The distribution of S under H_0 is obtained by Monte Carlo simulation (the χ^2 approximation is not valid here). The threshold associated to a size $\alpha = 0.01$ is $T_h = 95.10$. As $S = 15331.55 > T_h$, we reject H_0. The p-value (probability under H_0 of obtaining a value at least as extreme) is less than 2e-16 (Matlab double precision), thus there is very strong evidence against H_0.

For Hypothesis 2, the minimal value $S = 4862.16$ is obtained for $\lambda = 2.55e - 005$. It means that an element will move to an upper/lower level every $\frac{1}{\lambda} = 39216$ years on average (the distribution between time events indeed follows an exponential distribution with this parameter). The quantities π and E associated to this best fitted value are given in Tables S2-S3, respectively. The distribution of S under H_0 is obtained by Monte Carlo simulation (the χ^2 approximation is not valid here). The threshold associated to a size $\alpha = 0.01$.is $T_h = 192.27$. As $S = 4862.16 > T_h$, we reject H_0. The p-value (probability under H_0 of obtaining a value at least as extreme) is 0.0001.

For Hypothesis 3, the minimal value $S = 984.92$ is obtained for $\lambda = 5e - 006$. It means that an element will move to an upper/lower level every $\frac{1}{\lambda} = 200000$ years on average (the distribution between time events indeed follows an exponential distribution with this parameter). The quantities π and E associated to this best fitted value are given in Tables Tables S2-S3, respectively. The distribution of S under H_0 is obtained by Monte Carlo simulation (the χ^2 approximation is not valid here). The threshold associated to a size $\alpha = 0.01$ is $T_h = 211.25$. As $S = 984.92 > T_h$, we reject H_0. The p-value (probability under H_0 of obtaining a value at least as extreme) is 0.0006.

Probability models

Let π be the probability that an element found in Châtelperronian levels VIII-X is intrusive from Protoaurignacian level VII. This probability is unknown and we assume that it is distributed a priori from a uniform distribution over the interval [0,1]. Given that 0 out of 287 Dufour bladelets intruded and 0 out of 2800 unretouched bladelets intruded, if we apply the Bayes theorem (1), the posterior probability of π now follows a Beta distribution of parameters a=1, b=3088. Consequently, over a new set of 47 items (the total number of ornaments found in levels VII and VIII-X), the number of items that are intrusive from VII into VIII-X follows a Beta-binomial distribution of parameters n=47, a=1 and b=3088, whose probability mass function evaluated at k is given by

$$\frac{\Gamma(n+1)}{\Gamma(k+1)\Gamma(n-k+1)} \frac{\Gamma(a+k)\Gamma(n+b-k)}{\Gamma(a+b+n)} \frac{\Gamma(a+b)}{\Gamma(a)\Gamma(b)}$$

where Γ is the Gamma function. The probability mass function is represented in Figure S3a for the given parameters. It puts most of its mass at zero and the probability that more than 1 item is intrusive is <0.01.

We can carry out the same analysis using the Levallois flakes to assess the probability that items found in Châtelperronian levels VIII-X are displaced from underlying Mousterian levels XI-XII. Let π be the probability of moving from levels XI-XII to VIII-X. Given that 0 over 23 Levallois flakes have moved, the posterior probability of π follows a Beta distribution of parameters a=1, b=24. Consequently, over a set of 31 items (the total number of Neandertal teeth found in levels XI-XII and VIII-X), the number of items that have moved follows a Beta-binomial distribution of parameters n=31, a=1, b=24. The probability mass function is represented in Figure S3b. The probability that more than 7 items have moved is < 0.01.

For a set of 26 items (the total number of dated samples from levels VII and VIII-X), the number of items that are intrusive follows a Beta-binomial distribution with the same parameters as in the ornaments case except for n=26. The probability mass function is represented in Figure S3c. It puts most of the mass at 0, and the probability that more than 1 item is intrusive is <0.01.

Using a different approach, we can also assess the implications for the personal ornaments and the Neandertal teeth derived from accepting that the 38% anomalously young results obtained for Châtelperronian levels VIII-X reflect stratigraphic intrusion instead of incomplete sample decontamination. For the 39 personal ornaments, the probability that any one is intrusive is 0.38 with a 95% confidence interval of [0.18, 0.62]. Taking the higher limit of the interval (the most favorable for the disturbance hypothesis), the probability that all 39 are intrusive is 0.62^{39}, or 6e-9 (and, for a threshold of 1%, the maximum number of ornaments that could have been displaced under these probabilities is 31). By the same token, the probability that the 29 Neandertal teeth found in the Châtelperronian levels are all displaced can be calculated as 0.62^{29}, i.e., 8e-7 (and, for a threshold of 1%, the maximum number that could have been displaced under these probabilities is 24). As discussed above, however, such a level of disturbance should also be reflected in the distribution of the diagnostic stone tools, but it is not.

References

1. Bernardo JM, Smith AFM (2000) Bayesian Theory (Wiley and Sons, New York).
