# Supplementary figures and images for: The Reality of Neandertal Symbolic Behavior at the Grotte du Renne, Arcy-sur-Cure, France
Source: PLoS One. 2011 Jun 29;6(6):e21545. doi: 10.1371/journal.pone.0021545 (PMC3126825; doi:10.1371/journal.pone.0021545)

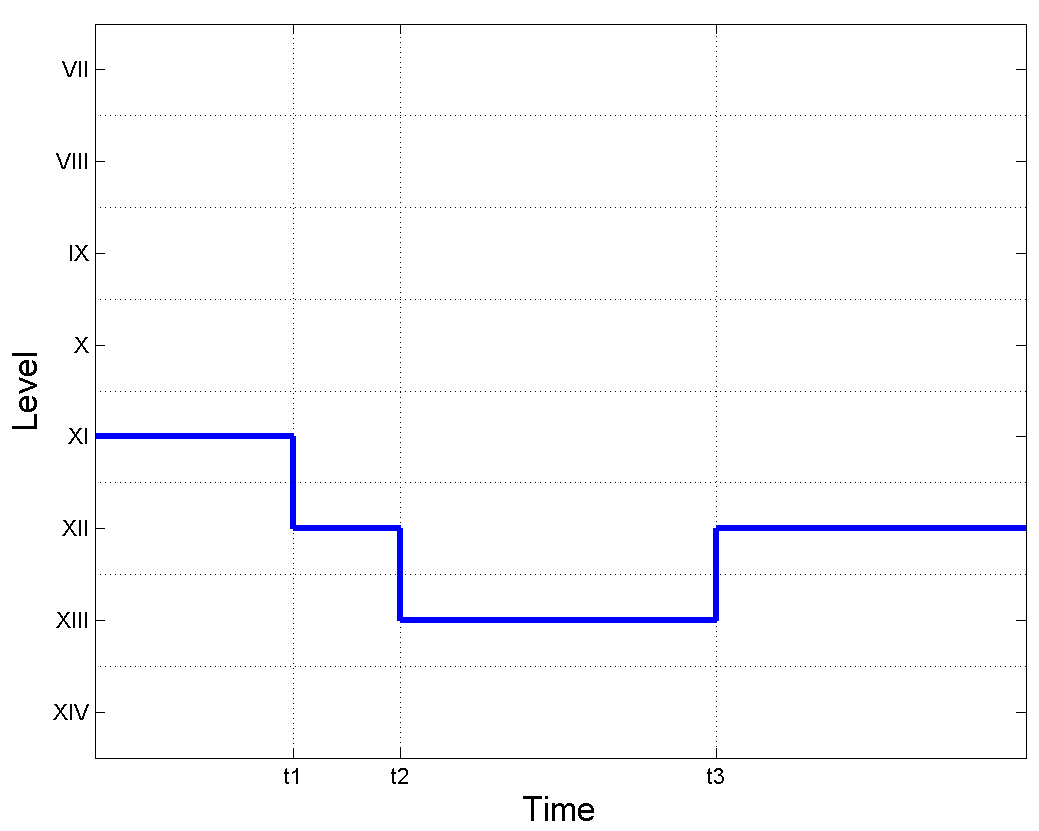

Supplement: Figure S1 — Realization from the continuous time model, for an object starting in level XI. (TIFF) [file pone.0021545.s002.tiff]

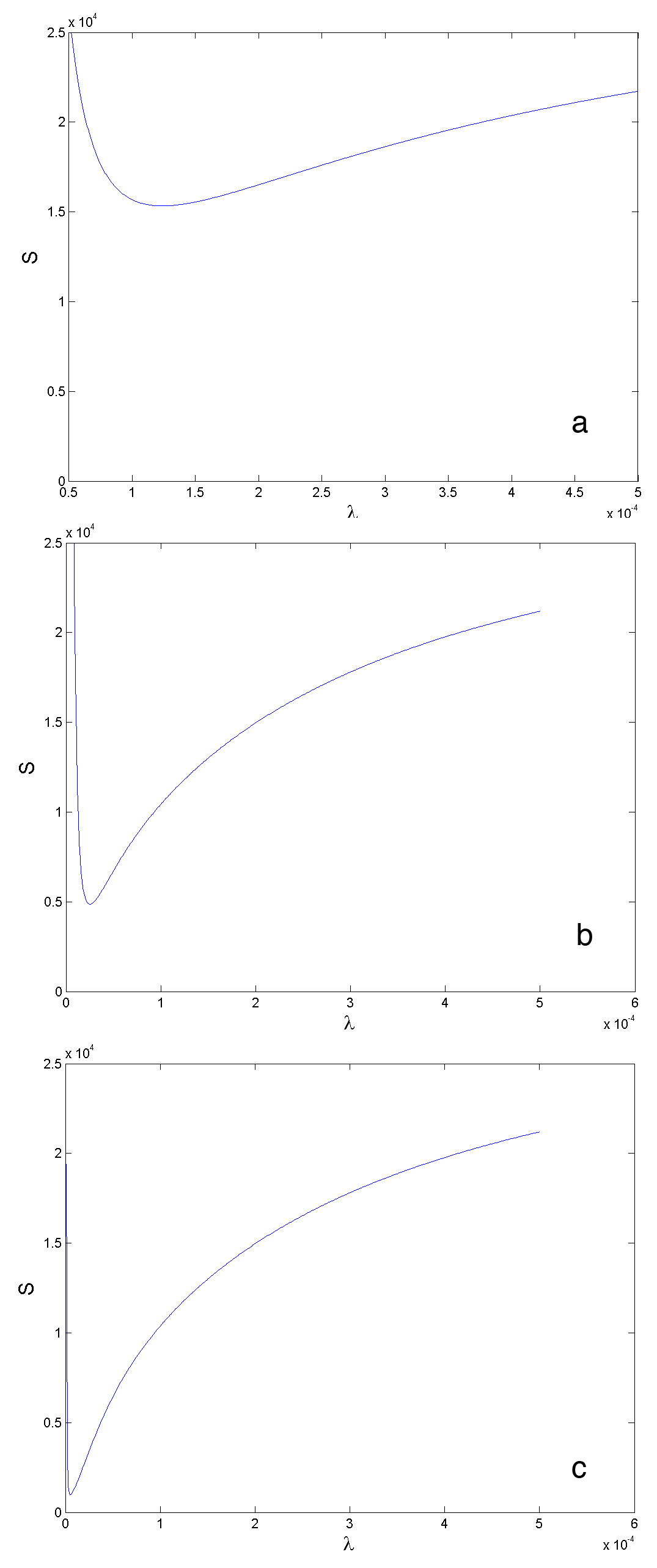

Supplement: Figure S2 — a: S in function of λ for Hypothesis 1; b: S in function of λ for Hypothesis 2, c: S in function of λ for Hypothesis 3. (TIF) [file pone.0021545.s003.tif]

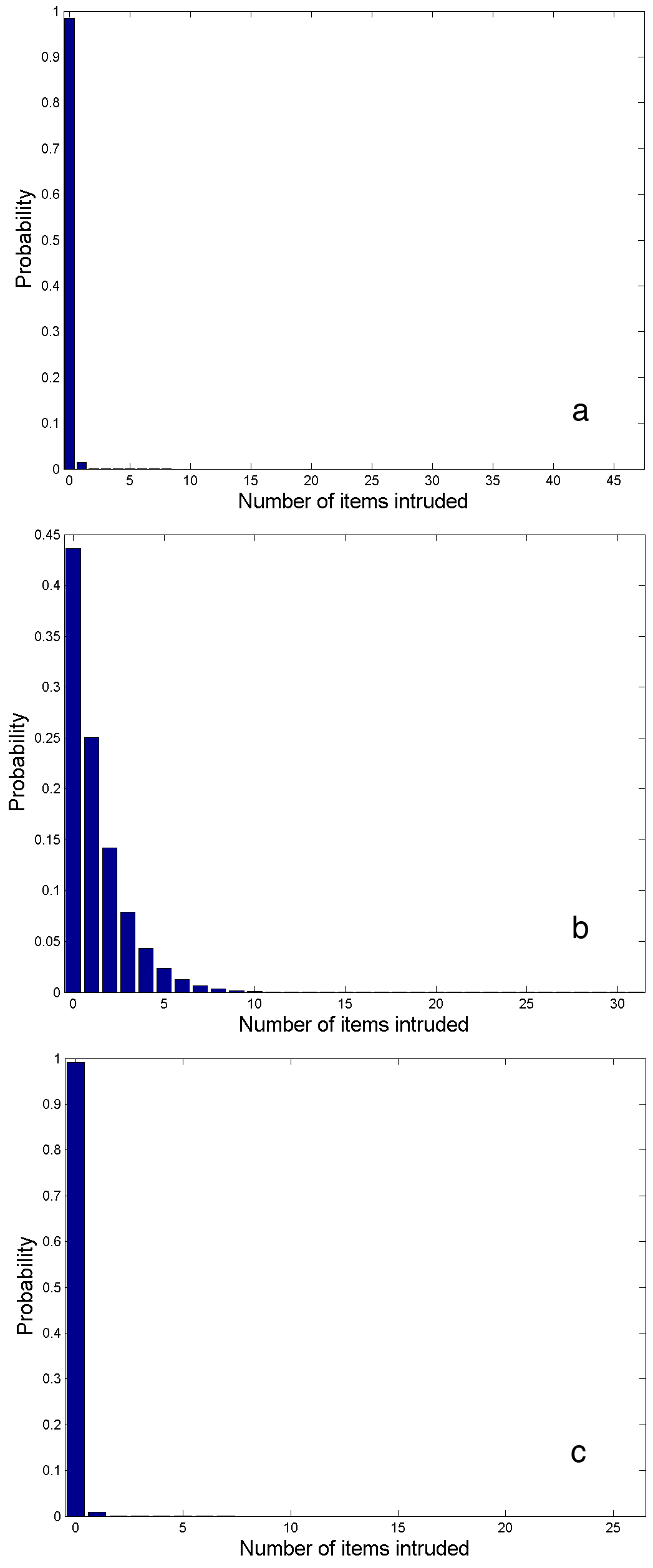

Supplement: Figure S3 — a: probability that k ornaments over a set of 47 (the total number of ornaments from levels VII and VIII–X) are intrusive, given the number of intruded Dufour bladelets and unretouched bladelets; b: probability that k Neandertal teeth over a set of 31 (the total number of Neandertal teeth from levels XI–XII and VIII–X) have moved from levels XI–XII to levels VIII–X, given the number of Levallois flakes that have moved; c: probability that k samples over a set of 26 (the total number of dated samples from levels VII and VIII–X) are intrusive, given the number of intruded Dufour bladelets and unretouched bladelets. (TIF) [file pone.0021545.s004.tif]
