# Supplementary material for: The Reality of Neandertal Symbolic Behavior at the Grotte du Renne, Arcy-sur-Cure, France
Source: PLoS One. 2011 Jun 29;6(6):e21545. doi: 10.1371/journal.pone.0021545 (PMC3126825; doi:10.1371/journal.pone.0021545)
Supplement: Table S1 — Predicted initial distribution of finds for the different tested hypotheses. (DOC) [file pone.0021545.s005.doc]

**Table S1**. Predicted initial distribution of finds for the different tested hypotheses.

| Levels | Ornaments | Pigments | Worked bone | Neandertal teeth | Dufour bladelets | Châtelperron points (a) | Levallois flakes | Unretouched bladelets | Convergent sidescraprers |
| --- | --- | --- | --- | --- | --- | --- | --- | --- | --- |
| For Hypothesis 1 | | | | | | | | | |
| VII | 47 | 1459 | 255 | 0 | 287 | 0 | 0 | 2800 | 0 |
| VIII | 0 | 0 | 0 | 0 | 0 | 30 | 0 | 0 | 0 |
| IX | 0 | 0 | 0 | 0 | 0 | 67 | 0 | 0 | 0 |
| X | 0 | 0 | 0 | 0 | 0 | 286 | 0 | 0 | 0 |
| XI | 0 | 0 | 0 | 30 | 0 | 0 | 9 | 0 | 139 |
| XII | 0 | 0 | 0 | 1 | 0 | 0 | 14 | 0 | 2 |
| XIII | 0 | 0 | 0 | 0 | 0 | 0 | 3 | 0 | 1 |
| XIV | 0 | 0 | 0 | 3 | 0 | 0 | 0 | 0 | 2 |
| For Hypothesis 2 | | | | | | | | | |
| VII | 47 | 39 | 70 | 0 | 287 | 0 | 0 | 2800 | 0 |
| VIII | 0 | 146 | 27 | 1 | 0 | 30 | 0 | 0 | 2 |
| IX | 0 | 286 | 17 | 3 | 0 | 67 | 0 | 0 | 28 |
| X | 0 | 1183 | 139 | 25 | 0 | 286 | 0 | 0 | 105 |
| XI | 0 | 5 | 1 | 1 | 0 | 0 | 9 | 0 | 4 |
| XII | 0 | 4 | 1 | 1 | 0 | 0 | 14 | 0 | 2 |
| XIII | 0 | 0 | 0 | 0 | 0 | 0 | 3 | 0 | 1 |
| XIV | 0 | 0 | 0 | 3 | 0 | 0 | 0 | 0 | 2 |
| For Hypothesis 3 | | | | | | | | | |
| VII | 8 | 39 | 70 | 0 | 287 | 0 | 0 | 2800 | 0 |
| VIII | 8 | 146 | 27 | 0 | 0 | 30 | 0 | 0 | 2 |
| IX | 2 | 286 | 17 | 0 | 0 | 67 | 0 | 0 | 28 |
| X | 29 | 1183 | 139 | 0 | 0 | 286 | 0 | 0 | 105 |
| XI | 0 | 5 | 1 | 30 | 0 | 0 | 9 | 0 | 4 |
| XII | 0 | 4 | 1 | 1 | 0 | 0 | 14 | 0 | 2 |
| XIII | 0 | 0 | 0 | 0 | 0 | 0 | 3 | 0 | 1 |
| XIV | 0 | 0 | 0 | 3 | 0 | 0 | 0 | 0 | 2 |
