# Supplementary material for: The Reality of Neandertal Symbolic Behavior at the Grotte du Renne, Arcy-sur-Cure, France
Source: PLoS One. 2011 Jun 29;6(6):e21545. doi: 10.1371/journal.pone.0021545 (PMC3126825; doi:10.1371/journal.pone.0021545)
Supplement: Table S2 — Expected final values E (rounded) associated to the best fitted λ for the different tested hypotheses. (DOC) [file pone.0021545.s006.doc]

**Table S2**. Expected final values *E* (rounded) associated to the best fitted λ for the different tested hypotheses.

| Levels | Ornaments | Pigments | Worked bone | Neandertal teeth | Dufour bladelets | Châtelperron points | Levallois flakes | Unretouched bladelets | Convergent sidescraprers |
| --- | --- | --- | --- | --- | --- | --- | --- | --- | --- |
| For Hypothesis 1 | | | | | | | | | |
| VII | 15 | 452 | 79 | 1 | 89 | 39 | 1 | 868 | 6 |
| VIII | 13 | 404 | 71 | 3 | 80 | 57 | 1 | 776 | 11 |
| IX | 9 | 291 | 51 | 4 | 57 | 70 | 3 | 559 | 18 |
| X | 6 | 172 | 30 | 6 | 34 | 72 | 4 | 331 | 24 |
| XI | 3 | 85 | 15 | 6 | 17 | 62 | 5 | 164 | 27 |
| XII | 1 | 36 | 6 | 6 | 7 | 44 | 5 | 69 | 25 |
| XIII | 0 | 13 | 2 | 5 | 3 | 26 | 4 | 26 | 19 |
| XIV | 0 | 4 | 1 | 3 | 1 | 14 | 3 | 8 | 12 |
| For Hypothesis 2 | | | | | | | | | |
| VII | 30 | 86 | 52 | 1 | 180 | 14 | 0 | 1758 | 3 |
| VIII | 14 | 211 | 45 | 3 | 82 | 45 | 0 | 805 | 13 |
| IX | 3 | 417 | 48 | 7 | 20 | 98 | 1 | 199 | 36 |
| X | 1 | 585 | 67 | 12 | 3 | 140 | 3 | 34 | 53 |
| XI | 0 | 273 | 32 | 6 | 0 | 65 | 7 | 4 | 26 |
| XII | 0 | 74 | 9 | 2 | 0 | 17 | 9 | 0 | 8 |
| XIII | 0 | 14 | 2 | 1 | 0 | 3 | 5 | 0 | 3 |
| XIV | 0 | 2 | 0 | 2 | 0 | 0 | 2 | 0 | 2 |
| For Hypothesis 3 | | | | | | | | | |
| VII | 8 | 49 | 66 | 0 | 259 | 3 | 0 | 2532 | 0 |
| VIII | 8 | 155 | 31 | 0 | 26 | 32 | 0 | 255 | 5 |
| IX | 5 | 352 | 29 | 0 | 1 | 83 | 0 | 13 | 32 |
| X | 24 | 985 | 114 | 3 | 0 | 238 | 1 | 0 | 88 |
| XI | 3 | 112 | 13 | 24 | 0 | 26 | 9 | 0 | 13 |
| XII | 0 | 10 | 2 | 4 | 0 | 1 | 12 | 0 | 3 |
| XIII | 0 | 1 | 0 | 1 | 0 | 0 | 4 | 0 | 1 |
| XIV | 0 | 0 | 0 | 3 | 0 | 0 | 0 | 0 | 2 |
