# Supplementary material for: The Reality of Neandertal Symbolic Behavior at the Grotte du Renne, Arcy-sur-Cure, France
Source: PLoS One. 2011 Jun 29;6(6):e21545. doi: 10.1371/journal.pone.0021545 (PMC3126825; doi:10.1371/journal.pone.0021545)
Supplement: Table S3 — Values π associated to the best fitted λ for the different hypotheses. (DOC) [file pone.0021545.s007.doc]

**Table S3**: Values $\pi$ associated to the best fitted $\lambda$ for the different hypotheses.

|  | | | Ending level | | | | | | | |
| --- | --- | --- | --- | --- | --- | --- | --- | --- | --- | --- |
|  |  | VII | | VIII | IX | X | XI | XII | XIII | XIV |
| For Hypothesis 1 | | | | | | | | | | |
| Starting level | VII | 0.31 | | 0.28 | 0.20 | 0.12 | 0.06 | 0.02 | 0.01 | 0.00 |
|  | VIII | 0.22 | | 0.24 | 0.22 | 0.16 | 0.09 | 0.05 | 0.02 | 0.01 |
|  | IX | 0.14 | | 0.19 | 0.21 | 0.19 | 0.14 | 0.08 | 0.04 | 0.02 |
|  | X | 0.08 | | 0.13 | 0.17 | 0.19 | 0.17 | 0.13 | 0.08 | 0.04 |
|  | XI | 0.04 | | 0.08 | 0.13 | 0.17 | 0.19 | 0.17 | 0.13 | 0.08 |
|  | XII | 0.02 | | 0.05 | 0.09 | 0.14 | 0.18 | 0.20 | 0.18 | 0.14 |
|  | XIII | 0.01 | | 0.03 | 0.06 | 0.10 | 0.16 | 0.21 | 0.23 | 0.21 |
|  | XIV | 0.01 | | 0.02 | 0.04 | 0.07 | 0.13 | 0.20 | 0.26 | 0.28 |
| For Hypothesis 2 | | | | | | | | | | |
| Starting level | VII | 0.63 | | 0.29 | 0.07 | 0.01 | 0.00 | 0.00 | 0.00 | 0.00 |
|  | VIII | 0.22 | | 0.48 | 0.22 | 0.06 | 0.01 | 0.00 | 0.00 | 0.00 |
|  | IX | 0.06 | | 0.21 | 0.45 | 0.21 | 0.06 | 0.01 | 0.00 | 0.00 |
|  | X | 0.01 | | 0.06 | 0.21 | 0.43 | 0.21 | 0.06 | 0.01 | 0.00 |
|  | XI | 0.00 | | 0.01 | 0.06 | 0.21 | 0.43 | 0.21 | 0.06 | 0.01 |
|  | XII | 0.00 | | 0.00 | 0.01 | 0.06 | 0.22 | 0.42 | 0.22 | 0.06 |
|  | XIII | 0.00 | | 0.00 | 0.00 | 0.01 | 0.07 | 0.23 | 0.44 | 0.23 |
|  | XIV | 0.00 | | 0.00 | 0.00 | 0.00 | 0.02 | 0.09 | 0.31 | 0.57 |
| For Hypothesis 3 | | | | | | | | | | |
| Starting level | VII | 0.90 | | 0.09 | 0.00 | 0.00 | 0.00 | 0.00 | 0.00 | 0.00 |
|  | VIII | 0.09 | | 0.83 | 0.09 | 0.00 | 0.00 | 0.00 | 0.00 | 0.00 |
|  | IX | 0.00 | | 0.09 | 0.82 | 0.09 | 0.00 | 0.00 | 0.00 | 0.00 |
|  | X | 0.00 | | 0.00 | 0.09 | 0.81 | 0.09 | 0.00 | 0.00 | 0.00 |
|  | XI | 0.00 | | 0.00 | 0.01 | 0.09 | 0.81 | 0.09 | 0.01 | 0.00 |
|  | XII | 0.00 | | 0.00 | 0.00 | 0.01 | 0.09 | 0.80 | 0.09 | 0.01 |
|  | XIII | 0.00 | | 0.00 | 0.00 | 0.00 | 0.01 | 0.10 | 0.80 | 0.10 |
|  | XIV | 0.00 | | 0.00 | 0.00 | 0.00 | 0.00 | 0.01 | 0.11 | 0.88 |
